# From Autonomy to Integration, From Integration to Dynamically Balanced Integrated Co-existence: Non-aging as the Third Stage of Development

**DOI:** 10.3389/fragi.2021.655315

**Published:** 2021-03-25

**Authors:** Lev Salnikov, Mamuka G. Baramiya

**Affiliations:** ^1^ SibEnzyme US LLC, West Roxbury, MA, United States; ^2^ AntiCancer, Inc., San Diego, CA, United States

**Keywords:** aging, senescence, multicellularity, carcinogenesis, reontogenesis, immunological tolerance, epigenetic

## Abstract

Reversible senescence at the cellular level emerged together with tissue specialization in *Metazoans*. However, this reversibility (ability to permanently rejuvenate) through recapitulation of early stages of development, was originally a part of ontogenesis, since the pressure of integrativeness was not dominant. The complication of specialization in phylogenesis narrowed this “freedom of maneuver”, gradually “truncating” remorphogenesis to local epimorphosis and further up to the complete disappearance of remorphogenesis from the ontogenesis repertoire. This evolutionary trend transformed cellular senescence into organismal aging and any recapitulation of autonomy into carcinogenesis. The crown of specialization, *Homo sapiens*, completed this post-unicellular stage of development, while in the genome all the potential for the next stage of development, which can be called the stage of balanced coexistence of autonomous and integrative dominants within a single whole. Here, completing the substantiation of the new section of developmental biology, we propose to call it Developmental Biogerontology.

“*It is not birth, marriage, or death, but gastrulation which is truly the most important time in your life*.” (Wolpert, 1991).

## Introduction

One of the central dogmas of unidirectional phylogenesis/ontogenesis consistently realized in evolution is a steady decrease of tissue-specific regenerative potential (*epimorphosis*), implementation of which requires recapitulation of early ontogenetic (embryonic) stages of development and expansion of autonomous cell potential. This is natural, because the entire second, post-unicellular stage of evolution was aimed at stabilization of multicellularity by limiting of autonomy, formation of specialized tissues and complication of integration processes. The “payment” for this achievement, which had undoubted evolutionary advantages–the conquest of new niches and improvement of all forms of life and mechanisms of adaptation, was the constant limitation of cellular autonomy in the interest of increasingly complex integrative dominants. As a result, there is a preserved but epigenetically blocked pathway for continuous and full quantitative and qualitative self-renewal of tissues, organs and functions. In other words, we have paid for reach the top of the current stage of development with inevitable involution, aging, aging associated diseases and mortality (Salnikov and Baramiya, 2020).

It is very important to understand that, in principle, there are no special genes and pathways for aging. These are the very mechanisms that ensure our functioning as a single integrated highly specialized whole. In other words, the way we exist makes us old. Yet this does not mean that we cannot change the described developmental pattern. However, any attempts to repeat the embryonic stages of development and autonomy in postnatal ontogenesis lead to carcinogenesis (disintegrating growth-DG). Consequently, the essence of the next, possible stage of *Homo sapiens*’ self-directed development lies in the systemic “consensus”, or dynamic balance of autonomous and integrative dominants within a single whole. As a result - the implementation of autonomous programs within the integrated whole, without disintegrating processes and with constant, complete and unlimited self-renewal of tissues, organs and functions.

## Ontogenesis and Its “Cost”

The main sign of aging is insufficient quantitative and qualitative/functional recovery of cell and tissue damage/deficiency accumulated over time. Thus, aging and aging related pathologies are a direct consequence of the non-compensability of losses (NB! not consequence of the uncompensated losses) that always accompany any functioning. Currently, antagonistic pleiotropy principles are the most commonly accepted explanation for aging (Williams, 1957). Our viewpoint on the functional division of the multicellular genome does not contradict it, but also provides a new understanding of this theory. We believe that pleiotropic properties are possessed not by individual genes acquired in evolution, but by their large group, which creates multicellularity as a specialized biological system and is united in the integrative part (IntG) of the cellular genome. In other words, the phenomenon of genes pleiotropy can always be described by the transition from symbiotic relations at the beginning of development, to parasitic ones at the end. It is this transition that occurs in multicellular organisms when their organismic organization passes from the necessary symbiotic relationships to parasitic ones (Salnikov and Baramiya, 2020). The resources required for improved integrative/organismal functions are taken away from the core autonomous functions of cells, represented by the THG portion of the genome. It is necessary to separate the cellular and organismal zones of regulation. The processes occurring exclusively in the organismal regulation zone and in its interests based on the IntG part of the genome leave the cellular one to cope with the consequences of this process within the limits of the decreasing capabilities of the THG part. It is important to understand here that, ultimately, the sequential limitation of autonomy in ontogenesis with each cycle of this limitation leads to gradual underproduction/“truncation” of function/functionality, including those specialized functions, for implementation of which autonomy is limited. The advantage gained by the IntG part of the genome, plays a major role from the evolutionary point of view, where it is important how developed the main functional systems of the organism will be by the time it reaches fertility. An advantage gained at the critical moment and paid for by suppressing of autonomous and regenerative potential in the future - it is this the IntG pleiotropy. It is this steady increase of the integration “functional tax” accompanied by the simultaneous epigenetic limitation of autonomy (freedom of maneuver) both in phylogenesis and ontogenesis that is the essence and the root cause of aging inherent in the very nature of genome functioning of highly integrated multicellularity.

Since the main developmental implementation mechanism is based on the predominance of IntG, which leads to a decrease in THG functionality, aging is also an integral part of ontogenesis. The definition of functional age in postnatal ontogenesis as the ratio of the functional activity of IntG/GHG genes is also associated with this. For successful development, the IntG part of the genome must from both upregulation of its genes and epigenetic downregulation of THG. Most likely, the functional predominance of IntG during ontogenesis is mediated by repressive methylation of THG, as evidenced by some experimental data (Garagnani et al., 2012) showing the increase in epigenetic shutdown of genes that we attribute to HG, is best related to the chronological age.

We assert that ontogenetic aging proper starts with epigenetic blocking of true stemness - cell pluripotency (the onset of gastrulation) and then continues by blocking of limited stemness - multipotency (the end of gastrulation). However, this does not mean that this is where functional and, consequently, biological aging of the organism begins. Functional aging begins since exceeding the critical value of the “functional tax” on HG, which reduces the vital adaptive resource for the cells and regenerative capabilities of the organism as a whole.

## “Wounds That do Not Heal” or Carcinogenesis as Unfinished Somatic Embryogenesis/Remorphogenesis

Like aging, carcinogenesis is a payment for multicellularity. The cells that give rise to cancer are immortal. The HeLa line has been maintained for seven decades, without any signs of degradation. In fact, we observe them as a unicellular' culture. Thus, the conclusion is obvious - elimination of the integration burden caused by the unidirectional ontogenetic program operation makes it possible to avoid the “payment” for its implementation. However, malfunction of intercellular control and integrity for the sake of autonomy and non-aging when the stimulus for cell division is produced only by the cells itself, results in carcinogenesis with inability to return/redifferentiate, escape from external control, loss of tissue-specific functionality, blockage of division control. So what is the solution?

Evolutionary trends revealed by the analysis of comparative and developmental immunology data demonstrate an inverse correlation between the ability to regenerate damaged/lost body parts and the development of an advanced immune system (Thouveny and Tassava, 1998; Harty et al., 2003; Mescher and Neff, 2005; Godwin and Brockes, 2006). More primitively organized *Metazoans* that rely solely on innate immunity have a greater regenerative potential. Compared to lower vertebrates such as amphibians and teleost fishes, which are able to completely regenerate many parts of the body, mammals have limited regenerative potential. To explain this difference, it has been postulated that the loss of regenerative potential in mammals is associated with the maturation of their immune system compared to lower vertebrates Julier et al., 2017; Uygur and Lee, 2016; Godwin and Rosenthal, 2014; Eguchi et al., 2011; Aurora and Olson, 2014; Bertolotti et al., 2013; Vitulo et al., 2017a; Kishi et al., 2012; Wilgus, 2007; Porrello et al., 2011).

Summarizing many other data, we conclude that the following is necessary for a successful tissue-specific epimorphic regeneration:

1. Proliferation of dedifferentiated cells with the formation of blastema, i.e. recapitulation of early ontogenetic stages of development (Gordon and Brockes, 1988; Morasso et al., 1996; Mullen et al., 1996; Carlson et al., 1998; Leask and Abraham, 2004; Vinarsky et al., 2005; Joetham et al., 2007; Lévesque et al., 2007; Rae et al., 2007; Satoh et al., 2008; Jopling et al., 2010; Campbell et al., 2011; Kubin et al., 2011; Satoh et al., 2011; Seifert et al., 2012; Godwin et al., 2013; Petrie et al., 2014; Cahill et al., 2017; Simkin et al., 2017; Vitulo et al., 2017a; Alibardi, 2020).
2. Engaging the Wnt signaling pathway and pluripotency/cellular reprogramming factors, including the so-called oncogenes (Fausto et al., 1986; Fausto, 1991; Staal et al., 2008; Maki et al., 2009; Monaghan et al., 2009; Lin et al., 2010; Neff et al., 2011; Boulter et al., 2012; Knapp et al., 2013; Looso et al., 2013; Sousounis et al., 2013; Stewart et al., 2013; Takeo et al., 2013; Godwin and Rosenthal, 2014; Hutchins et al., 2014; Raspopovic et al., 2014; Rivlin et al., 2014; Hesse et al., 2015; Kumar et al., 2015; Casey et al., 2016; Casey et al., 2017; Sarig and Tzahor, 2017; Vitulo et al., 2017b; Franco et al., 2018; Bywater et al., 2020; Shoffner et al., 2020; Xu et al., 2020; Ye et al., 2020)
3. Immunological tolerance through down-regulation (up to complete disappearance) of HLA-A, B, and -C and up-regulation of HLA-G and transformation of conventional effectors of the immune response into regulatory cells that provides the morphogenetic function of the immune system through active tolerance and stimulation of growth processes in renewing tissues (Maisel et al., 1998; Varda-Bloom et al., 2000; Veeneman et al., 2001; Krishnadasan et al., 2002; Shigematsu et al., 2002; Kubo et al., 2004; Brunetti et al., 2005; Hawrylowicz and O’Garra, 2005; Hemler, 2005; Ito et al., 2005; Taams et al., 2005; Ghiringhelli et al., 2006; Mak and Saunders, 2006; Mescher and Neff, 2006; Yamazaki et al., 2006; Yang et al., 2006; Collison et al., 2007; Joetham et al., 2007; Tiemessen et al., 2007; Staal et al., 2008; Corthay, 2009; Linfert et al., 2009; Sheng et al., 2009; Zoller, 2009; Lin et al., 2010; Perdiguero et al., 2011; Wang et al., 2010; Liu et al., 2011; Perdiguero et al., 2011; Petersen et al., 2011; Boulter et al., 2012; Rinkevich and Rinkevich, 2012; Tang et al., 2012; Burzyn et al., 2013; McHedlidze et al., 2013; Reinke et al., 2013; Takeo et al., 2013; Aurora et al., 2014; Diefenbach et al., 2014; Lavine et al., 2014; Li et al., 2014; Sadej et al., 2014; Weirather et al., 2014; Zordan et al., 2014; Artis and Spits, 2015; Castiglioni et al., 2015; Saito et al., 2015; Seubert et al., 2015; Berditchevski and Odintsova, 2016; Kudira et al., 2016; Ali et al., 2017; Hui et al., 2017; Krueger et al., 2017; Li and Hua, 2017; Mescher et al., 2017; Schiaffino et al., 2017; Simkin et al., 2017; Vannella and Wynn, 2017; Li et al., 2018; Schaper and van Spriel, 2018; Zacchigna et al., 2018; Zeng et al., 2018; Abnave and Ghigo, 2019; Li et al., 2019; Kew et al., 2020; Li et al., 2020).
4.  In highly specialized mammals, including humans, they exist in two cases: during embryogenesis and carcinogenesis (syn. somatic embryogenesis). However, the outcome in each of these cases is different.
5. How the obtained experimental data is interpreted depends largely on which “paradigmatic glasses” are used to view these data. Extensive review (Wong and Whited, 2020) summarizes important parallels between wound healing, epimorphic regeneration, and solid tumors. If one gets rid of the enemy-thinking view of carcinogenesis, then the conclusion will be unambiguous. In particular, the mechanisms that are carried out in postnatal ontogenesis as carcinogenesis initially debut as a variant of epimorphosis (syn. somatic embryogenesis). Cancer does not mislead the immune system at all, but the immune system itself reacts to the recapitulation of early ontogenetic programs in accordance with its morphogenetic function. However, this cooperation with the transformation of conventional effectors of the immune system into regulatory cells, leads to an active tolerance or regulatory facilitation reaction (Voisin, 1987) in highly specialized amniotes with a developed adaptive immune system. Only after that, the processes of tissue-specific regeneration are transformed into DG. On the contrary, dedifferentiation and an increase in cell autonomy with the formation of embryonic blastema in anamniotes with poorly developed/undeveloped adaptive immune system, which are at the stage of phylogenesis, at which cell autonomy is not only acceptable, but also a necessary component of their ontogenesis, this process leads to epimorphosis but not to carcinogenesis. The totipotent/pluripotent embryo is protected from the immune surveillance system by the same transformation mechanisms of conventional immune cells into regulatory cells at the mother-fetus interface and by a multinucleated nondividing syncytium (Trowsdale and Betz, 2006; Piao et al., 2015) It is the development in immunoprivileged conditions that makes it possible to form not a tumor, but a mature fetus. Only somatic cells that recapitulate early ontogenetic stages of development and at the same time are deprived of this immunoprivilege carry out this program in the form of carcinogenesis. The standard regenerative module during regeneration works within the program of relatively rigid-specific determination, and the level of tissue-specific differentiation is determined by the principle of submitting the interests of single units to the interests of the entire organism (IG-integrating growth). The active tolerance reaction ensures stability of this particular program. In the case of reontogenesis, that is, the recapitulation of morphogenetic modules of early ontogenesis (embryonic development program) with a specific tendency to autonomy, the same interactions ensure the stability of this particular inverse vector (unlimited expanding the potential of individual units - DG) that is detrimental to the entire organism. In other words, the immune response “bluntly” supports a growth program, which is launched when an absolutely critical or relative deficiency of tissue/functions occurs; regardless of whether the regenerative (within differentiation) or embryonic (with dedifferentiation and progressive autonomy) module works currently. Regulatory immune impacts in the absence of immunoprivilege blocks the possibility of returning to the state of differentiation, and it is from this moment that what began as epimorphic regeneration is transformed into DG. It is this makes somatic embryogenesis incomplete and turns the area of potential epimorphic regeneration into “wounds that do not heal” (Dvorak, 1986). Generation a full-fledged morphogenetic field in postnatal organs by re-creation of immunoprivileged status through complete immunological tolerance to the markers of the embryonic pathway (the so-called tumor-associated antigens) has oncostatic and differentiating properties and can prevent the transformation of reontogenesis into carcinogenesis. Many reports support this concept (Whisson, 1967; McKinnell et al., 1969; Mintz and Illmensee, 1975; Pierce and Wallace, 1971; Pierce et al., 1982; Pierce et al., 1987; Webb et al., 1984; Weaver et al., 1997; Coleman et al., 1993; Li et al., 2003; Lee et al., 2005; Gootwine et al., 1982; Park et al., 2017; Shvemberger, 1987; Nancy et al., 2012; Tong et al., 2018; Negishi et al., 2018; Zhao et al., 2017; Hyde and Schust, 2016; Farjadian et al., 2018; Amiot et al., 2011; Lin and Yan, 2018; Derynck and Weinberg, 2019; Curri and Bagehawe, 1967; Claser et al., 1965; Unkelles et al., 1974; Park et al., 2017).

It might be possible to eliminate the emerging cancer by killing it through the immune system. Meanwhile, this not eliminate but exacerbate the deficiency of tissues and functions, which will again lead to spontaneous reprogramming; in other words, it will trigger the mechanisms of epimorphic regeneration, which will turn into cancer again and again in the absence of immunoprivilege. The cancer eradication paradigm plays a major role in the palliative care of patients. While we are confident that this paradigm will never eliminate the cause of cancer, it will only reduce the cancer death probability. However, when one tries to eradicate cancer, rejuvenation will inevitably end, because spontaneous reprogramming which then turns into cancer, is an attempt by any living matter to renew itself. Full-scale reprogramming (spontaneous or induced) that never turns into cancer does not have a fully-fledged biological alternative for simultaneously solving two main problems: eliminating both cancer and aging. They cannot be solved separately, because the solution to these problems is the same. Briefly, it can be summarized as follows: in trying to eliminate aging, we will always “call for” cancer. Trying to kill cancer, we will always be destined to aging.

## From Geroprotective Tactics to Anti-Aging Strategy

The essence of geroprotection is to dampen the involution as much as possible-to delay the onset and to slow down progression of involution and age-related pathologies. Geroprotection has several main features that fundamentally distinguish it from anti-aging:

1. Geroprotection does not change the unidirectionality (formation → growth → involution → death) of ontogenesis/developmental vector;
2. It does not affect the root cause of aging, but individual signs and mechanisms associated with aging;
3. Geroprotective effects are relatively compensatory and transient.

In short, everything that does not correct the developmental vector is called geroprotection or elimination of consequences (such as the use of stem cells, correctly called progenitor cells, since they are not carriers real stemness - toti/pluripotency; calorie restriction, fasting, mimicking fasting, heterochronic blood and therapeutic plasma exchange, young plasma, secretome-based intervention, stem cell niche updating, NAD+, Resveratrol, Rapamycin (rapalogs), Metformin, Senolytics Oxytocin, Alk5i, Curcumin, ISRIB, so-called “epigenetic drugs”).

The essence of anti-aging is to eliminate the root cause of aging and, as a result age-related diseases. This requires a change in the unidirectionality of ontogenesis, in other words, an adjustment of the development vector.

We cannot always remain non-aging at the cellular level, since such agelessness is a function of autonomy, which being constant and unlimited come into conflict with specific functions of specialized structures, which is a function of integrativity, and invariably leads to DG. Consequently, the essence of the next (third) post-multicellular stage of *metazoans* development lies in the systemic developmental consensus/”reconciliation” of autonomous and integration dominants.

As a result - implementation of autonomous programs within the framework of an integrated whole without “sliding down” into DG on the one hand, and into irreversible involution on the other, which is equally inevitably fraught with loss of functionality. This will lead to complete and unlimited self-renewal of tissues/organs and functions, in other words, in non-aging at the organismal level. It is important to understand that aging, as a part of the developmental program cannot be avoided by influencing the individual mechanisms through which it is implemented. Only a program can bypass the program by modifying the unidirectional vector of development. Since we cannot “reverse” ontogenesis, this process is possible only within the framework of modification of the unidirectional developmental vector through its looping and corresponding changes in the principles of genome functioning. In our opinion, the only fundamental way of implementation of this complex developmental consensus is balanced coexistence of autonomous and integrative dominants, is what we figuratively call “to remember and accept ourselves” - to remember the prenatal self in the postnatal and through restoration of the immunological memory to accepted yourself as your own. There is the only way to achieve this to protect somatic embryogenesis from any types of the immune response, including active (regulatory) tolerance. In other words, the solution is in absolute immunological tolerance to antigens associated with early stages of embryonic development, for which the adult organism does not have memory. This is the essence of the proposed solution, to which we have lead in our previous publications (Baramiya, 2000; Baramiya et al., 2020).

## Discussion

Aging is a process and a consequence of the processes of a steadily increasing limitation of the ability of full-fledged tissue-specific self-renewal at all levels of organization and non-compensability of losses of cells/tissues/functions (always accompanying any functioning), due to the sequential realization in the development of the central dogma of unidirectional phylogenesis/ontogenesis of *metazoan* - a steadfast epigenetic restriction of cellular autonomy in the interests of increasingly complex integration dominants. This turns their functional (specialized) part from a cellular symbiont into a cellular parasite, limits lifespan and leads to death due to involutive extinction of functions, failure of regulatory homeostatic mechanisms, emergence of endogenous disorders and an increased susceptibility to exogenous factors.

Involution and loss of functionality are only the consequences of aging. Aging per se is an inability to completely restore functionality, and it is inherent in the very nature of the organization (genome functioning) of highly specialized multicellularity. Until this understanding is realized and accepted, we will eliminate the consequences but not the root cause.

Living matter has an existential “urge” to conquer habitat niches by improving mechanisms and forms of adaptation, including through self-renewal. This is the only essence and “goal” of evolution. A full-fledged tissue-specific renewal is possible only through recapitulation of the early stages of ontogenesis, that is, through endogenous systemic reprogramming. Systemic natural reprogramming with zeroing of epigenetic and metabolic load and age in ontogenesis occurs twice: the first time in the prenatal period as a result of fertilization and leads to fetal formation, the second time in postnatal ontogenesis and leads to carcinogenesis. We should finally understand a clear pattern: any genes and signal pathways that inhibits senescence takes part in the potentiation of carcinogenesis; any genes, signal pathways that suppresses carcinogenesis stimulates senescence. Therefore, neither aging nor carcinogenesis can be eliminated without “reconciliation” of these processes. There are no bad genes (signaling pathways), and what we consider their insufficient or excessive functioning, or an error in turning them on and off, is in fact often quite natural developmental variants that can lead to undesirable consequences for us. To overcome them, we must simply use the other options available and not blindly suppress certain undesirable effects for us. Once again - the program of unidirectional ontogenesis can be overridden only by an alternative program, but not by purposeful suppression or stimulation of certain developmental variants, which in the absence of a three-dimensional picture of interactions and interdependencies of all signal networks is a random search with a local, but not systemic desired result and sometimes with very negative consequences. Whereas the “reconciliation” of autonomy with integrativity through the establishment of their dynamic balance will allow to get away from the functional pleiotropy of the IntG while maintaining the integrity of the symbiont with the simultaneous preservation of the functioning autonomy within the integrated whole.

There is reason to believe that *Homo sapiens* have not yet reached the pinnacle of evolution. Therefore, completing here our series of publications (Baramiya, 1988; Baramiya, 2000; Baramiya, 2018; Baramiya and Baranov, 2020; Baramiya et al., 2020; Salnikov and Baramiya, 2020) on the theoretical substantiation of a new section of developmental biology, we propose to call it Developmental Biogerontology.

## Dedication Statement

‘This article is dedicated to my coauthor and friend, Dr. M.G. Baramia, who passed away suddenly and untimely. I am certain that his ideas and the direction in gerontology to which he dedicated his scientific life will be continued in the research of his colleagues.’ - Dr. Lev Salnikov.

## Abbreviations

HG, housekeeping genes; AHG, autonomizing housekeeping genes; GHG, growth housekeeping genes; THG, total housekeeping genome(AHG + GHG); IntG, integrative genes; IG, integrating growth; DG, disintegrating growth.

## Glossary

HG: housekeeping genes
AHG: autonomizing housekeeping genes
GHG: growth housekeeping genes
THG: total housekeeping genome(AHG + +GHG)
IntG: integrative genes
IG: integrating growth
DG: disintegrating growth
Integrating Growth: a submission of potency of single cells composing an organism to the development program and functions of the whole organism
Disintegrating Growth: a priority of extension potency of single cells over the development program and functions of the whole organism.
